# Peer support for disadvantaged parents: a narrative review of strategies used in home visiting health interventions in high-income countries

**DOI:** 10.1186/s12913-020-05540-8

**Published:** 2020-07-23

**Authors:** Per Kåks, Mats Målqvist

**Affiliations:** Uppsala Global Health Research on Implementation and Sustainability (UGHRIS), Department of Women’s and Children’s Health, Uppsala University, Uppsala, SE-75185 Sweden

**Keywords:** Peer support, High-income settings, Developed countries, Family, Vulnerable populations, Minority groups, Breast feeding, Community health workers, Doulas, Mentors, Social support, Child health, Maternal health, Family health

## Abstract

**Background:**

Disparities in health persist even in high-income countries, and healthcare systems do not reach disadvantaged families as needed. A number of home-visiting interventions in high-income countries offering peer support for parents have been implemented to bridge the gaps in health in a cost-effective way. The lack of standard for intervention design has however resulted in a large variety of the strategies used. The objective for this article is to conduct a review of peer support home visiting interventions for parents and children in high-income countries, aiming to assess the strategies used, their outcomes and the challenges faced by peer supporters.

**Methods:**

Relevant articles published in English between January 2004 and August 2019 were identified using PubMed, and reference lists were reviewed to identify additional articles. Studies were included if they reported on individual peer support health interventions, delivered at home to socioeconomically disadvantaged parents in high-income countries. Nineteen studies were found that met the inclusion criteria, and data were extracted on study characteristics, intervention design and outcomes. Data on intervention design was characterized iteratively to generate overarching categories of strategies used in the programs.

**Results:**

Most studies used healthcare facilities for recruitment, even when the interventions were not delivered by the formal healthcare system. The strategies used to engage supported parents included (1) connection in the form of emotional support, relationship building and matching for background, (2) flexibility in regards to content, intensity, location and mode of contact, and (3) linking through referrals and facilitation of other contacts. A number of significant quantifiable improvements could be demonstrated. Due to large heterogeneity of outcomes, meta-analyses were not viable. Peer supporters experienced challenges with involving other family members than the supported parent as well as with finding their role in relation to other support structures.

**Conclusions:**

Peer support delivered as home visiting interventions have been used for hard-to-reach parents in a variety of high-income contexts and for a multitude of health concerns. Overall, despite variation in intervention design, the strategies employed followed common themes and were generally well received.

## Background

The status of socioeconomic vulnerability as a negative determinant of maternal and child health has become evident in a number of quantifiable outcomes [1, 2]. These include a higher risk of giving birth to premature babies and babies with low birth weight among disadvantaged mothers [3], lower rates of breastfeeding initiation and shorter duration [4, 5], as well as higher incidences of postpartum depression [6, 7]. Due to the fact that the early parts of a child’s life is formative in both a positive and a negative sense, suboptimal conditions in the perinatal period can have long-lasting effects on health, behavior and parent-child relationships [8–10].

Preventive healthcare aimed towards parents and children can reduce morbidity and mortality [11], as well as reduce overall healthcare costs [12]. This care does not, however, reach every family equally and socio-economically disadvantaged parents are often hard to reach for healthcare systems in high-income countries [13, 14]. In order to reach the most vulnerable parents and children, home visiting programs have been successfully implemented alongside regular health services in several low- and middle-income countries. Some of these have demonstrated reductions in child mortality, particularly with case management of ill children, improvements in immunization status, decline in underweight children, improvements in breastfeeding rates and reductions in prevalence of perinatal depression, but data on long-term effects is generally lacking [15, 16]. Similarly, home visiting with nurses or midwives have had measurable positive effects in high-income countries [17–19]. Parts of the home visiting procedure, such as social and practical support, does not however require extensive medical training. Because of this, home visiting programs have increasingly been employing community non-professionals as peer support home visitors. The evidence for these is mixed, and while comparisons with nurse-delivered interventions generally favors the latter, peer support home visits may offer some benefits over no intervention at all [20–22].

Peer supporters can in theory be both less expensive than medical professionals and act as role models for supported parents if they share a common medical or social background. Due to enormous differences in social context, replicating successes with home visiting models from the global south in high-income settings may require adaptation of both strategies to initiate contact, to engage and to retain supported parents. A number of home-visiting programs have already been trialed in high-income settings, but there is a lack of standardization. Previous reviews of the literature on peer support home visits for disadvantaged parents have generally focused on evaluating the effectiveness of the interventions, with only occasional and relatively brief overviews on the methodology of their delivery [23, 24]. Displaying the variations in how home visits are delivered can highlight the various ways that the delivery of such interventions can be both consciously adapted to different contextual needs, and ways in which fidelity to methods of implementation can be evaluated. The latter is important as consistency in program delivery may impact the consistency in its content, and thereby in the expected outcomes as well. The aim of this review is to provide such an overview by summarizing the main strategies for initiating contact and strategies to engage parents through peer support interventions in high-income countries, targeting disadvantaged families. Furthermore, the review will compile outcomes from such interventions as well as the challenges faced by peer supporters during the processes.

## Methods

### Terminology

There are various terms used to denote home-visiting peer support workers, and the lack of standardized terminology mirrors the lack of a standard in intervention delivery. The well-used concept of *doulas*, referring to women providing emotional support and physical comfort during labor, has been expanded to *community doulas*, providing support not only during birth but also in perinatal activities through home visits [25]. Health-promoting interventions in American Hispanic communities have employed *promotoras*, working on promoting healthy habits among families in the target communities [26]. Interventions in South Africa have been led by *mentor mothers*, striving to empower women and promote maternal and child health in disadvantaged areas [27, 28]. Other words describing similar home visitors include *community health worker*, *peer supporter* and *lay supporter*. Throughout this review, the term *peer supporter* was used to denote all types of similar home visitors.

### Search strategy

The procedure outlined by Green et al. [29] was used for compilation of findings. Papers included in this review were original studies evaluating peer support interventions for parental and child health. This sample was chosen to reflect all the formative phases of parenthood and children’s development, spanning from the prenatal period to later stages of childhood, as peer support interventions often span over several phases of development. The heterogeneity in regards to child age and development phase in this review reflects some of the earlier literature on the topic [16, 30]. Studies for inclusion were identified in two ways. A literature search in PubMed was conducted by the first author for primary studies published during a 15-year period from January 2004 to August 2019. The terminology used to denote peer supporting community workers varies widely between studies and the search strategy was adapted to accommodate for this. The search string, used to search titles and abstracts, was *[(“peer support” OR “lay support” OR “community health worker” OR “mentor” OR “promotora” OR “doula”) AND (“maternal” OR “paternal” OR “mother” OR “father” OR “parent”)]*. The first author screened citations from the search for eligibility through reading titles, abstracts or full text articles, using Rayyan QCRI software, and in the case of uncertainty regarding if papers fit the set criteria they were discussed with the second author.

### Inclusion and exclusion criteria

In order to qualify for inclusion in this review, studies had to:

- focus on health-related peer support interventions for parents or parents to-be
- be implemented by peer supporters, i.e. with limited formal medical training
- target parents belonging to socioeconomic disadvantaged groups
- be implemented in high-income countries in accordance with the 2018 World Bank definition
- be delivered as home visiting programs
- be written in English
- be primary studies
- be peer-reviewed

Studies were excluded if:

- the social support was provided through groups rather than individual mentoring
- the interventions were focused on support for children or adolescents themselves rather than their parents
- they lacked clear descriptions of strategies for contact, content of the interventions, strategies to engage or challenges faced by peer supporters

### Appraisal

Each article was critically appraised by the first author using the assessment questions for study quality outlined by The Critical Appraisal Skills Programme (CASP) [31]. These questions include evaluation of appropriateness of research question, study design, statistical methods and interpretation, conflicts of interest and relevance. The included articles were appraised using quality checklists corresponding to their study design, ensuring that each study fulfilled the minimum criteria without ascribing them scores.

### Data extraction and analysis

From the included articles, data was extracted on main study characteristics (author, year, location, target groups, health topic, measures), intervention design (point of contact, setup, methods for delivering intervention content) and findings (outcomes for parents and children, challenges for peer supporters). Data on main study characteristics were summarized in table form. The data on methods for delivering intervention content were categorized by first cataloguing all explicit descriptions in the methods sections of each included paper on how each intervention was delivered, in terms of both proactive and reactive definitive actions and responses to participants’ practical and emotional needs. For the studies that presented results from interviews with peer supporters, a similar cataloging was also done of qualitative descriptions of how the content was delivered in the result sections of each paper. The complete list of descriptions of intervention delivery was reviewed by reading and re-reading and emergent themes in methodology were identified inductively, compared and characterized iteratively by the first author to identify conceptual overarching strategies to engage target groups. The validity of the analysis was confirmed by review of the articles by the second author. A meta-analysis was not carried out due to large variations in both intervention content, statistical analysis and diversity in measured outcomes. The synthesis of the data from all studies is presented narratively below.

## Results

**Fig. 1:**
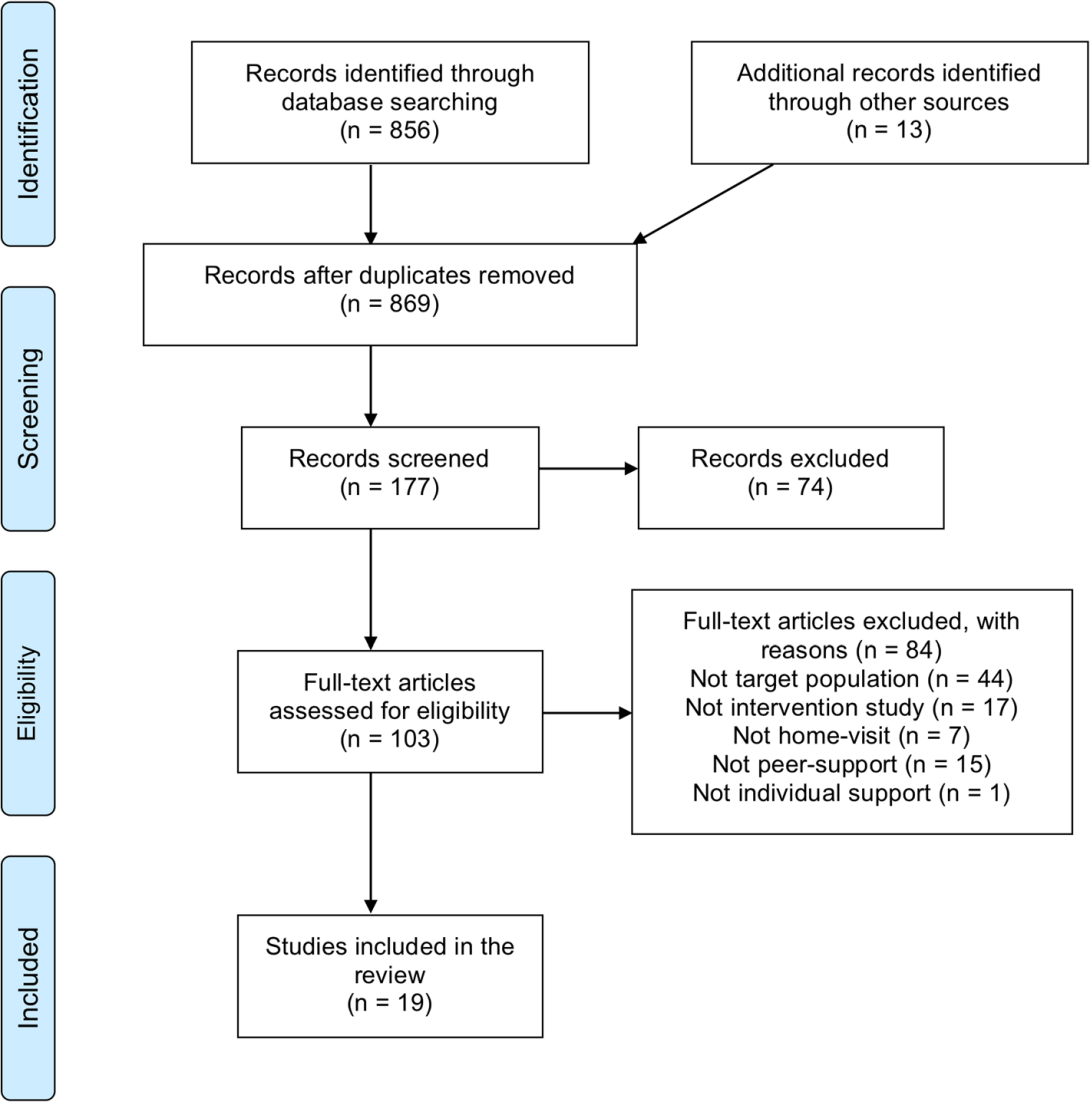
The process of study selection

A total of 856 articles were identified from the original search, whereof 10 met the inclusion criteria. Through scanning of the reference lists of the included articles an additional nine articles matching the criteria were obtained. The full presentation of the selection process is shown in Fig. 1. The final 19 articles, representing 17 interventions, are presented in Table 1. One of the interventions had three corresponding articles, by Hans et al., [32] Edwards et al. [33] and Thullen et al. [34].

**Table 1:** Included studies

Authors	Reference	Setting	Health topic	Type	Sample	Intervention setup
Barlow A, Mullany B, Neault N, Compton S, Carter A, Hastings R, Billy T, Coho-Mescal V, Lorenzo S, Walkup J T	Effect of a paraprofessional home-visiting intervention on American Indian teen mothers’ and infants’ behavioral risks: A randomized controlled trial. Am. J. Psychiatry 170, 83–93 (2013)	South West United States	Parent-child interaction, perinatal health	Quantitative	Pregnant women in gestational week ≤32, aged 12–19 and living in Native American reservation area (*n* = 322).	43 highly standardized sessions, delivered through prengnancy to 3 years after birth, initially weekly and later less frequently.
Bolton T A, Chow T, Benton P A, Olson B H	Characteristics associated with longer breastfeeding duration: An analysis of a peer counseling support program. J. Hum. Lact. 25, 18–27 (2009)	Michigan, United States	Breastfeeding	Quantitative	Pregnant women and women with child in breastfeeding age, and with low family income (*n* = 5067).	Monthly or more contacts from birth until 1 year after birth or to breastfeeding discontinuation.
Crespo N C, Elder J P, Ayala G X, Slymen D J, Campbell N R, Sallis J F, McKenzie T L, Baquero B, Arrendondo E M	Results of a multi-level intervention to prevent and control childhood obesity among Latino children: The Aventuras Para Niños study. Ann. Behav. Med. 43, 84–100 (2012)	San Diego, United States	Child nutrition	Quantitative	Hispanic mothers with a child in kindergarten (*n* = 808).	Weekly home visits for 7 months and phone calls every 6 months for 2 years.
Edwards R C, Thullen M J, Korfmacher J, Lantos J D, Henson L G, Hans S L	Breastfeeding and complementary food: Randomized trial of community doula home visiting. Pediatrics 132 Suppl 2, S160–6 (2013)	Unspecified area, United States	Breastfeeding, child nutrition	Quantitative	African American women pregnant in gestational week < 34, with low family income (*n* = 248).	10 weekly prenatal home visits, presence during birth. 12 weekly home visits for 3 postnatally.
Graffy J, Taylor J, Williams A, Eldridge S	Randomised controlled trial of support from volunteer counsellors for mothers considering breast feeding. BMJ 328, 26 (2004)	London, United Kingdom	Breastfeeding	Quantitative	Pregnant women in gestational week 28–36 and living in deprived area (*n* = 710).	One prenatal visit, postnatal contact by phone for unspecified time period, more home visits if needed.
Hans S L, Edwards R C, Zhang Y	Randomized controlled trial of doula-home-visiting services: Impact on maternal and infant health. Matern. Child Health J. 22, 105–113 (2018)	Illinois, United States	Breastfeeding, maternal mental health, perinatal child health	Quantitative	Pregnant women aged < 26 years and in gestational week < 34, in low-income families (*n* = 312).	Weekly visits during pregnancy and 3 months postnatally, as well as joining for medical appointments and during labour.
Hans S L, Thullen M, Henson L G, Lee H, Edwards R C, Bernstein V J	Promoting positive mother-infant relationships: A randomized trial of community doula support for young mothers. Infant Ment. Health J. 34, 446–457 (2013)	Unspecified area, United States	Parent-child interaction	Quantitative	African American women pregnant in gestational week < 34, with low family income (n = 248).	Weekly visits during pregnancy and post-partum by home-visitor and doula, from 6 weeks to 3 months post partum only home-visitor.
Ingram J	A mixed methods evaluation of peer support in Bristol, UK: mothers’, midwives’ and peer supporters’ views and the effects on breastfeeding. BMC Pregnancy Childbirth 13, 192 (2013)	Bristol, United Kingdom	Breastfeeding	Mixed	Mothers with children in breastfeeding age living in deprived areas (*n* = 14), peer supporters (n = 7) and maternity health workers (n = 5).	One antenatal contact, postnatal telephone contact for 2 weeks
Kenyon S, Jolly K, Hemming K, Hope L, Blissett J, Dann S-A, Lilford R, MacArthur C	Lay support for pregnant women with social risk: a randomised controlled trial. BMJ Open 6, e009203 (2016)	West Midlands, United Kingdom	Breastfeeding, maternal mental health, perinatal child health	Quantitative	Nulliparous and pregnant in gestational week < 28, with at least one social risk factor (*n* = 1324).	Visits with unspecified frequency from gestational week 28 to 6 weeks postpartum.
Lee E, Mitchell-Herzfeld S D, Lowenfels A A, Greene R, Dorabawila V, DuMont K A	Reducing low birth weight through home visitation: A randomized controlled trial. Am. J. Prev. Med. 36, 154–160 (2009)	New York, United States	Perinatal child health	Quantitative	Women who were pregnant or with child < 3 months old, with low income and risk of child maltreatment (*n* = 501).	Biweekly visits during pregnancy.
McLeish J, Redshaw M	‘We have beaten HIV a bit’: a qualitative study of experiences of peer support during pregnancy with an HIV Mentor Mother project in England. BMJ Open 6, e011499 (2016)	London, United Kingdom	HIV	Qualitative	Mothers with diagnosis of HIV, primarily immigrants from Sub-Saharan Africa (n = 6) and peer supporters (*n* = 6).	Visits with flexible frequency before, during and after pregnancy as needed.
Murphy C A, Cupples M E, Percy A, Halliday H L, Stewart M C	Peer-mentoring for first-time mothers from areas of socio-economic disadvantage: A qualitative study within a randomised controlled trial. BMC Health Serv. Res. 8, 46 (2008)	Belfast, United Kingdom	Perinatal child health	Qualitative	First-time mothers aged 16–30 years living in deprived area (n = 11) and peer supporters (*n* = 11).	Biweekly visits during pregnancy up to 1 year postpartum
Rotheram-Fuller E, Swendeman D, Becker K, Daleiden E, Chorpita B, Youssef M K, Rotheram-Borus M J	Adapting current strategies to implement evidence-based prevention programs for paraprofessional home visiting. Prev. Sci. 18, 590–599 (2017)	Los Angeles, United States	Breastfeeding, maternal mental health, maternal nutrition	Quantitative	Pregnant Hispanic and Korean/American-Korean mothers living in a low-income area (*n* = 101).	Biweekly visits for 8 weeks during pregnancy and 8 times over 6 moths postpartum.
Taverno Ross S E, Barone Gibbs B, Documet P I, Pate R R	ANDALE Pittsburgh: Results of a promotora-led, home-based intervention to promote a healthy weight in Latino preschool children. BMC Public Health 18, 360 (2018)	Pittsburgh, United States	Child nutrition	Quantitative	Hispanic mothers with children aged 2–5 years, living in low-income area and with generally low acculturation (*n* = 51).	10 weekly visits.
Thomson G, Crossland N, Dykes F	Giving me hope: women’s reflections on a breastfeeding peer support service. Matern. Child Nutr. 8, 340–353 (2012)	North West England, United Kingdom	Breastfeeding	Qualitative	Breastfeeding mothers living in deprived area (*n* = 47).	Visits with unspecified frequency during 8 weeks.
Thomson G, Dykes F, Hurley M A, Hoddinott P	Incentives as connectors: insights into a breastfeeding incentive intervention in a disadvantaged area of North-West England. BMC Pregnancy Childbirth 12, 22 (2012)	North West England, United Kingdom	Breastfeeding	Mixed	Breastfeeding mothers living in deprived area (n = 26 for qualitative data, *n* = 266 for quantitative) and peer supporters (n = 4).	Weekly visits from birth to 8 weeks postpartum.
Thullen M J, McMillin S E, Korfmacher J, Humphries M L, Bellamy J, Henson L, Hans S	Father participation in a community-doula home-visiting intervention with young, African American mothers. Infant Ment. Health J. 35, 422–434 (2014)	Unspecified area, United States	Perinatal child health	Mixed	African American women pregnant in gestational week < 34, with low family income (n = 248).	Weekly visits during pregnancy and up to 3 months postpartum.
Watt R G, Tull K I, Hardy R, Wiggins M, Kelly Y, Molloy B, Dowler E, Apps J, McGlone P	Effectiveness of a social support intervention on infant feeding practices: randomised controlled trial. J. Epidemiol. Community Health 63, 156–162 (2009)	London, United Kingdom	Child nutrition	Quantitative	New mothers with infants < 12 weeks old, belonging to non-professional occupational class (n = 312).	Monthly visits from 3 months to 1 year.
Williams C M, Cprek S, Asaolu I, English B, Jewell T, Smith K, Robl J	Kentucky Health Access Nurturing Development Services home visiting program improves maternal and child health. Matern. Child Health J. 21, 1166–1174 (2017)	Kentucky, United States	Perinatal child health	Quantitative	First-time mothers with at least two social risk factors (*n* = 4506).	Often weekly visits during pregnancy, lower frequence postnatally and up to 2 years after birth.

Eleven of the studies were designed as randomized controlled trials, three were qualitative studies, three were mixed method studies, one was a quantitative observational study and one was quasi-experimental. The quality of each article was not rated according to a scoring system, but all included articles were deemed to satisfy the minimum criteria outlined by the CASP quality checklists.

### Target groups

All included studies focused on disadvantaged groups of mothers, and none on fathers. The criteria for including participants varied, and three themes of assessing socioeconomic adversity emerged: personal vulnerability, disadvantaged demographic group and disadvantaged resident area. Several interventions followed more than one of these themes, targeting groups that experienced adversity in more than one sense.

#### Personal vulnerability

Personal vulnerability was usually in the form of low family income [32–37], even though only one of the studies reported recruitment based on an absolute income limit, defined as 200% of federal poverty level [37]. Another type of personal vulnerability was social risk, which included several types of socioeconomic and interpersonal risk factors. Kenyon et al. required one social risk factor for recruitment to their intervention in the U.K., and considered, among other things, participants to be eligible if belonging to the lowest income quintile, having housing problems such as temporary accommodation or struggling with written and spoken English [38]. Williams et al. required two risk factors for inclusion, with the main risk factors being inadequate income, receiving Medicaid and living with an unemployed partner [39].

#### Demographic groups

Recruitment by disadvantaged socioeconomic groups were based on ethnicity [32–35, 40–43] and age [32–35, 42]. The ethnic minority groups in question were Hispanics [40, 41, 43], Koreans [40], Native Americans [42] and African Americans [32–35]. McLeish and Redshaw used HIV diagnosis rather than ethnicity as a basis for recruitment, but the large majority of their participants were immigrant women with a Sub-Saharan African background [44]. When age was used to determine eligibility for participation, the age bracket of the supported mothers varied from 12 to 19 years in Barlow et al. [42] to up to 30 years in Murphy et al. [45]. One intervention recruited participants up to 22 years [32–34], and one had an age limit of 26 years [35].

#### Resident area

Other interventions determined eligibility primarily based on residence in a deprived area, but this could be difficult to separate from criteria of personal adversity or minority group status. Rotheram-Fuller et al. recruited women living in an area outside of Los Angeles, where practically all inhabitants were Hispanic or Korean and all had incomes below 200% of the federal poverty level [40]. Five interventions targeted women living in deprived areas in the U.K., without using any individual factors as criteria [45–49].

### Strategies to initiate contact

Five of the included interventions based the initial contact with parents on routine antenatal clinic visits where the parents were given information about the studies and were offered to participate [32–34, 38, 40, 45, 46]. Two of these also used a baseline interview at the antenatal clinic or through phone, for assessment of current experiences of pregnancy and eligibility [32–34, 40]. One of the studies described a peer supporter being present during an antenatal appointment to introduce the planned intervention program and inform the participants about its contents [33]. The study by Watt et al. [50] reported recruitment of mothers through postnatal baby clinics using a screening questionnaire to assess eligibility.

Another strategy to recruit participants was to make direct contact based on screening, referrals or word of mouth. In the study by Ingram [49], names of pregnant women in the area were collected weekly from local community midwives by the organization offering the peer support service, a strategy agreed on as suitable by all parts. The pregnant women were contacted by a peer supporter during the later stage of the pregnancy to offer a home visit or discuss other available support for breastfeeding. The same supporter also contacted the women within 2 d of discharge from the birth clinic to offer further support. Similar strategies were used in two other studies [35, 42] where young pregnant women were referred to the program by other maternal health intervention programs, health clinics, schools and public health departments. One of these studies also reported recruitment through word of mouth in the study area [42]. Both an antenatal service organization, a local hospital and a family health center was used to find potential study participants by Lee et al. [37]. Another study also relied on referrals to the intervention program, but did not specify who were responsible for those referrals [39]. Taverno Ross et al. [41] reported using promotoras who recruited participants in the study from their own social networks. This was done during community gatherings, through flyers and word of mouth. Eligibility screening of potential participants was conducted in-person or through phone. Another study [43] using a promotora-led intervention, initiated contact with parents to children in kindergarten through schools in the study area. The principal of each school gave permission to recruit parents directly on school grounds, through flyers sent home with the children and during presentations at the schools.

Three of the studies recruited study participants that already had an established contact with a peer support organization for evaluation of their experience or health-related habits [36, 44, 48]. All of these studies reported that initial contact with the organization was made by the parents after referral from healthcare professionals. In the study by Bolton et al. [36], this process was facilitated by the fact that the organization was sharing an office with the referring health clinic. Thomson et al. also reported using self-referrals from mothers, via distribution of posters and leaflets in community settings and clinics [47].

### Intervention setups

Focuses and contents of the interventions spanned over several topics and methods of delivery. Several of the studies featured complex interventions with more than one main focus within the area of maternal and perinatal child health. The evaluated quantitative measures of these studies included maternal mental health [35, 38, 40], birth outcomes [37, 39], use of pain medication during labor [35, 38], maternal-infant bonding [38, 39], feeding practices [35, 38, 40], immunization rates [38] and level of engagement in perinatal care [38]. Qualitatively, experiences of a peer support program [45] and participation of fathers [34] were explored.

The most frequent topic for the studies with a single focus was breastfeeding, where initiation rates, duration and exclusivity were evaluated quantitatively, [33, 36, 46, 49, 50] along with qualitative evaluations of experiences of receiving breastfeeding support programs [47–49]. Child nutrition and overweight prevention was the focus in three of the studies, with changes in BMI [41, 43], physical activity [41] and micronutrient intake [50] being reported. Two studies reported on how quantified measures of parent-child interaction and attitudes changed during interventions [32, 42]. The study by McLeish and Redshaw [44] was the only one having done a support intervention for mothers with HIV, which was evaluated through qualitative descriptions of the mothers’ experiences of the program.

The interventions varied in mode of contact, frequency and duration. All but two studies reported having a proactive approach towards the use of home visits, in the sense that visits were consistently planned in advance according to a schedule. An alternative to this approach, described by Ingram [49] and Graffy et al. [46], was to only plan one prenatal home visit and subsequently arrange more visits if the supported parents expressed a need for it, otherwise relying on phone-based support. A common frequency for home visits was consistent weekly face-to-face contact [32–35, 41, 48], though some studies focusing on perinatal health used weekly visits during pregnancy with lower frequency postnatally [39, 42]. Biweekly [37, 40, 45] and monthly visits [36, 43, 50] were used by three interventions. No clear correlation could be found between the frequency of contact and the areas of focus in the respective interventions.

The support was delivered over a period ranging from 3 weeks to over 3 y. Among those starting the home visiting programs during pregnancy and continued after birth, two ended the intervention 6 weeks after birth [38, 40], two after 8 weeks [47, 48], and one after 12 weeks [32–34]. Three of the interventions started during pregnancy and continued for one to 3 y after birth [39, 42, 45]. Three programs only had prenatal visits, two of which only relied on a single home visit with subsequent phone contacts after birth to support breastfeeding [46, 49], and one starting before a gestational age of 30 weeks and continuing until birth [37]. Some programs, all of which focused on feeding practices and nutrition, used only postnatal visits. These were conducted over a period of 10 w [41], 7 m [43], 9 m [50] and 1 y [36] respectively. One program, providing support for mothers both before and after birth, tailored the duration and intensity of the intervention to the individual needs of every supported mother [44].

### Strategies to engage target groups

Assessment and iterative characterization of the intervention designs used showed that a set of recurring operational methods could be identified. These methods could be classified into three conceptual strategies: (1) connection (emotional support, cultural/demographic matching, relationship building), (2) flexibility (intensity and duration, content, mode of contact, location), and (3) linking (referrals, facilitation of other contacts) (Table 2).

**Table 2:** Strategies used in the interventions

Study	Connection			Flexibility				Linking	
	Emotional support	Cultural/ demographic matching	Relationship building	Flexible intensity and duration	Flexible content	Flexible mode of contact	Flexible location	Referrals	Facilitation of other contacts
Barlow et al. (2013)		✓	✓				✓	✓	
Bolton et al. (2009)		✓	✓	✓		✓		✓	
Crespo et al. (2012)		✓							
Edwards et al. (2013)	✓	✓	✓			✓	✓		
Graffy et al. (2004)									
Hans et al. (2013)	✓	✓	✓			✓	✓		✓
Hans et al. (2018)	✓	✓					✓	✓	
Ingram (2013)									✓
Kenyon et al. (2016)	✓								✓
Lee et al. (2008)	✓	✓	✓				✓		
McLeish and Redshaw (2016)	✓	✓		✓		✓			
Murphy et al. (2008)	✓	✓		✓		✓		✓	
Rotheram-Fuller et al. (2017)	✓	✓			✓	✓			
Taverno Ross et al. (2018)		✓							
Thomson et al. (2012a)	✓					✓			
Thomson et al. (2012b)	✓	✓				✓		✓	
Thullen et al. (2014)	✓	✓				✓	✓		
Watt et al. (2009)	✓	✓				✓			
Williams et al. (2017)				✓	✓				✓

#### Connection

The majority of the interventions were described as using emotional support to engage parents in the programs [32–35, 37, 38, 40, 44, 45, 47, 48, 50]. This included non-practical guidance through challenges faced by the parents, such as inspiring hope and displaying non-judgmental acceptance while acting as mentors. In order to form connections between the home visitors and the parents most interventions also put an emphasis on the importance of a shared background. This was done not only in regards to experience of pregnancy and parenthood but also with a common culture or demographic belonging in mind. Peer supporters were often recruited on the basis of being able to speak a certain minority language [37, 40, 43] or belonging to the same ethnic group as the parents receiving support [32–34, 37, 42, 44]. Religious matching could also enhance emotional connection and experiences of support. In the study by McLeish and Redshaw [44], the shared religious faith between the peer supporters and the supported mothers was described as an asset, sometimes being used to reframe faith issues acting as a barrier to trust in medical treatment. The same study also described how a shared experience of immigration to the U.K. contributed to a sense of insight into the supported mothers’ socially difficult situation. Other studies recruited peer supporters mainly based on sharing demographic characteristics with the target groups, without necessarily specifying ethnicity or cultural belonging [35, 36, 41, 45, 47, 50]. The majority of the studies that did not report taking cultural or demographic backgrounds of the peer supporters into consideration evaluated interventions in areas with predominantly white populations [38, 39, 46, 48].

Another strategy for connections between supporter and parent was to use relationship-building to retain program participants and facilitate behavioral change. Four interventions reported on using development of rapport as a conscious strategy [32, 33, 36, 37, 42]. This rapport-building consisted of deliberate efforts to establish a trusting relationship between the supporter and the supported. However, in several studies the relationship was put forth as a consequence of the respective interventions rather than as a method to engage participants [34, 44, 45, 47, 48]. The relationship formed between supporter and supported were described in terms of ‘friendship’ [45, 47], ‘family’ [44] and ‘being on the journey together’ [48].

#### Flexibility

All but five studies gave descriptions of how flexibility was used as a tool to engage and retain participants. This flexibility could be found both in how the interventions were structured in regards to frequency and duration, in the contents of the support provided to each individual family, in the use of telephone as an alternative mode of contact with supported parents, and in how visits were carried out. Two interventions tailored the frequency of visits to the expressed needs of the participants [39, 45], and two customized the intervention in regards to both intensity and duration [36, 44]. Clear descriptions of individual tailoring of informational content was also provided for two interventions [39, 40], both of which used targeted content based on goals set up together with each family. One of these interventions, by Rotheram-Fuller et al., set up new goals at the end of every visit, evaluating the families’ abilities to reach them with two-week intervals [40].

Contact through telephone as a complement to home visits was used in three distinctly different ways. One way was to use regularly scheduled telephone contacts as the main mode of delivering support, with occasional home visits if a clear need was indicated [46, 49]. Secondly, phone contacts could be used to maintain scheduled contact after a period of face-to-face meetings, to reinforce the support and advice previously given [43]. The third way of using telephone contacts was to offer them as a flexible complement or alternative to face-to-face contact, when home visits were not feasible or when additional contact with the peer supporter was needed [32–34, 36, 40, 44, 45, 47, 48, 50]. A variant of this method was to keep a very high level of access to support by offering the possibility to reach the peer supporters through telephone 24 h a day [33]. Flexible availability was stressed by McLeish and Redshaw as a factor contributing to a sense of genuine emotional connection, allowing the relationship to take the form of a friendship [44].

Flexibility was also offered in regards to how and where face-to-face meetings were carried out. Several interventions had peer supporters who occasionally joined the supported mothers to clinical appointments as social support [32, 34, 35, 37]. These appointments consisted of regular antenatal maternal care, ultrasound examinations and postpartum visits [34, 35]. Two interventions also employed peer supporters who were present during labor and delivery as doulas, providing physical comfort, advocacy and emotional support to the mother [32–35]. Apart from company in clinical settings, face-to-face meetings could also take the form of transportation assistance to address crises that arose during the trial [42].

#### Linking

The final conceptual strategy used was to link to other services outside of the boundaries of the interventions. These were sometimes done as formal referrals to social services [45], midwives or other types of medical professionals such as frenotomy clinics [47], nutrition programs [36], primary care for immunization and mental health appointments [35, 36] or to unspecified healthcare services [42, 45]. The peer supporters could also help the supported families to find community resources for basic access to stable housing, food and benefits [38, 39]. Two interventions used a more passive approach to facilitating links, one by giving information about breastfeeding groups [49] and one by encouraging the mothers to make their own contact with local services [32].

### Quantitative outcomes

Fifteen of the studies reviewed provided quantified outcomes of their respective peer support interventions, presented below. A summary of primary outcomes and results can be found in Additional file 1.

Tracking parameters of neonatal and infant health, a large quasi-experimental study conducted by Williams et al. was able to show several significant improvements for families receiving interventions [39]. Preterm deliveries were lower compared to the control group (10.6% vs 13.7%, OR 0.74, 95% CI 0.61–0.88), along with a birth weight < 2500 g (7.2% vs 12.4%, OR 0.54, 95% CI 0.44–0.67). Similar improvements in birth weight was seen in the study by Lee et al. [37], with a close to halved incidence in the home visit intervention group (5.1% vs 9.8%, AOR 0.43, 95% CI 0.21–0.89). A significant effect on the incidence of low birth weight was however absent in two other studies [35, 38], one of which furthermore reported no effect on prematurity or cesarean section rates [35].

The interventions aiming to promote breastfeeding had varying degrees of success, with some studies showing higher breastfeeding initiation rates but none being able to show sustained effects over longer periods of time. Five studies reported no significant effects on breastfeeding from peer support or from introduction of incentives in a support program [38, 39, 46, 48, 49]. Edwards et al. [33] however saw significantly higher likelihood of initiation in the intervention group than in the control group (64% vs 50%), as well as higher breastfeeding rates after 6 w (29% vs 17%). This difference was no longer significant after 4 m, when the mothers were no longer receiving home visits. Furthermore, the intervention group had a lower rate of giving the infants complementary food at 6 w (6% vs 18%). Similar patterns were seen in the intervention by Hans et al. [35], with significant improvements in breastfeeding among supported mothers directly after birth (81% vs 74%, OR 1.67, 95% CI 0.91–3.03) but without sustained effects after 3 months despite continued home visits. Both these studies used doulas as peer supporters both prenatally, during birth and postnatally, which no other of the breastfeeding studies did. Another study [40] measured the correlation between the time peer supporters spent on various strategies and topics of discussion with mothers and the duration of their breastfeeding, reporting a significant correlation to discussing breastfeeding (*r* = .28) and parenting (*r* = .30). Other significant correlations were between the use of relaxation (*r* = .27) and attending (*r* = .24) as strategies and breastfeeding duration. Correlations were also observed by Bolton et al. [36] between breastfeeding duration and time of introduction of formula, where shorter duration was seen among those who introduced formula at day 1 (− 37.9 days among participants enrolled prenatally and − 49.1 days among those enrolled postnatally). Breastfeeding duration was also positively correlated to maternal age and previous breastfeeding experience.

None of the interventions that aimed towards improving child overweight rates, micronutrient intake or physical exercise resulted in overall significant improvements [41, 43, 50], but one intervention improved weight among the children with highest initial BMI [41]. The same intervention was able to improve child intake of fruits, vegetables, saturated fat and sugar, as well as reduce screen time and increase parent physical activity. In the study by Rotheram-Fuller et al. [40] maternal BMI was shown to be correlated to how much time the peer supporters spent discussing coping with depression, but no conclusions could be drawn regarding cause and effect.

Maternal mental health was assessed with the Edinburgh Postnatal Depression Scale (EPDS) in three studies [35, 38, 40]. Kenyon et al. reported reductions in mean EPDS scores (− 0.79 compared to control group) 8–12 weeks postnatally for women with two or more social risk factors, such as low age, language difficulties or lack of support from close family members. However, no effect was seen in the intervention group at large. In another study no effects could be seen neither at 3 weeks or at 3 months postpartum [35]. A negative correlation between addressing depression during home visits and depression outcomes (*r* = .27) was noted by Rotheram-Fuller et al. [40].

Regarding maternal somatic health, effects on pregnancy-induced hypertension (9.4% vs 17.5%, OR 0.51, 95% CI 0.42–0.60) and complications during delivery (1.6% vs 2.7%, OR 0.60, 95% CI 0.40–0.91) could be seen by Williams et al. when analyzing data from an intervention with over 4500 participants [39]. In the perinatal period the intervention-group parents were also more likely to adequately take part in prenatal care (73.6% vs 71.0%, OR 1.14, 95% CI 1.00–1.13) and have contact with a nutrition program (92.0% vs 88.2%, OR 1.57, 95% CI 1.28–1.93), and were less likely to be reported for child maltreatment (6.0% vs 11.0%, OR 0.53, 95% CI 0.43–0.65). Another study using both home visits and peer support during birth reported higher attendance in preparation classes before birth (50.0% vs 9.5%, OR 9.82, 95% CI 4.84–19.89) and lower use of pain medication during labor (71.8% vs 83.2%, OR 0.47, 95% CI 0.25–0.88) [35]. Significant improvements were also seen in this study in regards to safety behavior such as use of car seats and letting infants sleep on their backs.

Mothers of infants were reported by Hans et al. to have better parenting attitudes and behaviors after an intervention involving doulas, who supported the mothers prenatally, during labor and postnatally [32]. This set of improved practices involved positive engagement with the infants (Cohen’s d 0.17), response to distress (Cohen’s d 0.35) and parenting values with the child’s best in mind (Cohen’s d 0.24). The infants were also less likely to show visible upset during observations (Cohen’s d 0.24). However, all these effects faded over time after the intervention had ended. Mother-to-infant bonding was also significantly better among mothers receiving peer support when measured through a questionnaire at 8–12 weeks by Kenyon et al. [38]. Another study saw improvements in both parenting knowledge, self-efficacy and home safety attitudes while still receiving home visits at 12 months postpartum, but no significant effects on actual home safety practices [42].

The rate of attrition was reported for 9 of the interventions [32–35, 38, 41–43, 46, 48, 50]. The large differences in durations of the interventions were reflected in large variations in retention rates. The lowest retention rate at the end of the study, at 55% 3 years after the start of the intervention, was seen by Crespo et al. [43], and the highest at 96% was reported by Taverno Ross et al. [41] at 10 weeks after start of the intervention.

### Challenges faced by peer supporters

Qualitative descriptions were featured in three studies, regarding challenges experienced by peer supporters and supported parents during the intervention processes. In the study by Murphy et al. [45], one such challenge was difficulties in establishing initial contact and rapport with target mothers. Partly these difficulties were related to practicalities such as women not being home for the visits and not replying to initial text messages or phone calls. Other challenges included cultural barriers and low interest from parents due to lack of understanding of the role of the peer supporter, who sometimes were expected by parents to be a healthcare professional or social care worker. The study also reported initial uncertainty among peer supporters of what the job of the peer supporter entailed in regards to actual provision of health-related information, in contrast to pure social and emotional support. Such provision of information and advice could contradict advice from the parents’ extended family, hindering the peer supporters’ ability to build strong relationships with the parents marked by trust. In the study by Ingram [49] the uncertainty of the peer supporters’ role was initially experienced by the midwives working alongside them, but this uncertainty reduced during the duration of the intervention program.

Involvement of both parents and the extended family was approached differently in the interventions. Murphy et al. [45] reported negative experiences of peer supporters on the involvement of others, as health-related information from peer supporters could lead to conflicting advice from several sources, as previously mentioned, as well as interference with continuation of the program and diminished ability to communicate freely during the home visits. The study by Ingram [49] revealed difficulties by breastfeeding-promoting peer supporters in engaging partners in home visits when such attempts were made, mainly due to practical reasons such as home visits taking place during working hours. The same practical challenges of involving working partners of supporter mothers was reported by Thullen et al. [34], even though most partners were positive towards the intervention. This study furthermore highlighted the problem of a subset of young fathers seeing peer supporters as a reason to lower their own involvement in perinatal activities, as the peer support interventions decreased the mother’s need for social and practical support from partners.

Peer supporters in several of the studies also stressed the importance of cultural and social matching to provide optimal conditions for a good mentor-mentee relationship. Murphy et al. [45] reported this as crucial to the peer supporters’ ability to form close relationships, get a deeper understanding of the needs of the parents and to adapt the content of the interventions to suit these needs. The response to lack of cultural matching was generally to give information through pre-set agendas rather than individual tailoring to needs, reducing the personalization of the content.

## Discussion

Home-visiting peer support programs for disadvantaged parents often recruit participants through healthcare facilities, but sometimes also through more community-based methods. The strategies used to engage supported parents could be categorized into three overarching conceptual strategies: (1) connection in the form of emotional support, relationship building and matching for background, (2) flexibility in regards to content, intensity, location and mode of contact, and (3) linking through referrals and facilitation of other contacts. Although outcomes were mixed, improvements in several types of quantifiable outcomes could be demonstrated. Peer supporters sometimes struggled with finding their role and involving other family members, but the support was generally well received.

Several previous reviews of lay home-visiting programs aimed towards improving maternal and child health have been published. These have generally focused on evaluating the effectiveness of the interventions [16, 30], sometimes by evaluating cost-effectiveness [51], or by comparing effectiveness in high-income countries to that of low and middle income settings [52]. A few reviews have explored how home-visiting interventions for maternal and child health have been delivered [23, 24, 53], but to the authors’ knowledge this is the first review that thoroughly synthesizes methodology of delivery for peer support programs delivered as home visits for underprivileged parents in high-income countries. The categories of strategies used to deliver peer support that was found in the iteration in this review broadly reflect how previous literature has described the content of support programs for disadvantaged groups [54, 55]. In a qualitative study of experiences from the voluntary sector of working with hard-to-reach groups, Flanagan and Hancock found four facilitators in their analysis of strategies: ‘Treatment of clients - trust, respect’, ‘Flexibility’, ‘Partnership working’ and ‘User involvement’ [55]. The first three of these roughly resemble the overarching strategies in this review, but we did not see a theme of involving service users in the working process.

The articles included in this review varied in study design, statistical methods and sample size. As the main goal of this review was compilation of strategies used in interventions for recruiting and engaging supported parents, no studies were excluded on the grounds of sample size or methods. However, both sample size and social context of the interventions may affect the generalizability of the outcomes (i.e. ethnic minorities being studied). This holds true especially for the qualitative studies, where individual experiences may influence conclusions to a large degree.

As with any review, the strategies and outcomes of studies included risk being affected by a publication bias. As authors are more likely to publish findings displaying an improvement in the measured outcomes, the strategies used in the interventions as well as their outcomes may tend to reflect those of successful programs. There is also a risk of bias within the studies in regards to what is reported, as authors tend to report positive findings more often than negative. We therefore caution not to use the compilation of outcomes to draw conclusions of what can be expected in terms of results from future peer support interventions.

Peer support interventions for maternal and child health have a long history in low-income countries such as South Africa and Pakistan [15]. Systematic reviews have however demonstrated difficulties in replicating successes in high-income settings. One such review by Jolly et al. [52] highlighted the low success rates of breastfeeding peer support programs in high-income countries as compared with low-income countries, possibly due to the already existing support from public healthcare services in the former. The low success rates of breastfeeding interventions over time found by the studies included in this review is in line with this. Some of the included studies covering other topics were able to demonstrate positive results, but consideration has to be taken to the sometimes large number of outcomes measured.

Defining disadvantaged populations from single criteria may be difficult, as demonstrated by the fact that most studies included in this review had target populations with several simultaneous factors that made them vulnerable. Difficult economic situations overlapped with minority group belonging, unstable housing and employment, low maternal age and language difficulties. These multiple vulnerabilities result in a social and medical complexity with two aspects: disadvantaged populations may have a heightened need for care, but they can also be harder to reach by conventional social and health systems. What characterizes hard-to-reach groups has been explored in other reviews. Sokol et al. has described hard-to-reach populations as those whom healthcare and targeted interventions fail to reach due to cultural, environmental, demographic and individual reasons [56]. The majority of studies in this review had participants that could be classified as being selected primarily on demographic or individual criteria, but they may at the same time have fit into the cultural or environmental classifications. The participants in the study by McLeish and Redshaw exemplified this well, as they experienced challenging circumstances ranging from recent immigration and homelessness to religious and language barriers preventing effective adherence to healthcare advice [44]. Targeting populations with multiple reasons for being hard-to-reach may require more from intervention programs, but such populations may also be the ones with the largest need for support. This need for support further motivates the use of referrals to health care and social resources, as disadvantaged populations may need support in seeking out such services.

Most studies used healthcare facilities as a main point of initial contact, either by recruiting participants on-site or through referrals. This may result in well-grounded assessments of who is truly in need of participation in an intervention program, but it is also dependent on that an established contact with the healthcare system exists in the first place. Other studies had a more community-based recruitment process, using word of mouth, flyers, posters and recruitment through schools [41–43, 47]. This approach enables recruitment of individuals without established healthcare or social service contact, but it also makes the process of finding study participants somewhat less systematic.

It is important that the would-be supported parents understand what the peer support service entails, and the study by Edwards et al. was the only one that reported having a peer supporter on-site during recruitment to explain the contents of the program and answer questions [33]. This strategy is a way of building initial rapport before a decision to participate is made, and it could be a way of addressing challenges of establishing initial contact, such as those reported by Murphy et al. [45].

Peer support may have the ability to offer something that conventional healthcare systems sometimes lack, in the form of continuous emotional support and connection through a shared background and personal experience. Through this it can act as a complement rather than as an alternative to conventional care. Both emotional support and a shared background were reported as strategies by a majority of the studies reviewed, and several qualitative studies stressed how participants saw it as important to establish a deeper personal connection between the supporter and the supported [44, 47, 48]. This connection promoted retention and engagement of participants in the programs. It also led to more meaningful conversations which in turn opened up the flow of personal and practical information in both directions. The impact of the cultural difficulties reported by Murphy et al. [45] may hint towards a need for further research to explore to what extent cultural matching affects intervention outcomes and participant retention. Similarly, other factors such as frequency and timing of contact might be relevant for the supported parents’ decision to continue or discontinue their participation in interventions of this type, and these have to be explored further in future evaluations of peer-delivered home visiting programs.

The aim towards complementing rather than replacing existing support structures is also important in regards to the family. As described by Thullen et al. and Murphy et al., peer supporters’ advice and help could sometimes compete with that of partners and relatives, demonstrating a need for having a plan for involving others [34, 45]. The lack of certainty regarding how to relate to family members could perhaps be prevented through recruitment of previously supported parents as peer supporters, given that the program is not new. Such recruitment of former clients can potentially give the peer supporters a strong sense from the start of what their work should entail in order to provide the most benefit for the parents and their family.

Support programs should be attentive and adaptive to the needs of their participants. The flexible approach to location, mode of contact, content and intensity allowed for participants to shape the support they receive to maximize its benefits in many of the interventions, with only five studies reporting use of none of these strategies [38, 41, 43, 46, 49]. Especially one study, by Rotheram-Fuller et al., stressed the importance of not needing to replicate a manual with absolute fidelity, as this allowed for higher personalization and parental engagement [40]. The highest levels of flexibility of all interventions was arguably demonstrated by Edwards et al., where doulas were reachable by phone during 24 h a day [33]. Such level of commitment to the role as a peer supporter can be valuable for the creation of genuine relationships, but a consideration has to be made regarding what is justifiable to expect from supporters. Their status as employees or volunteers can also determine the level of personal flexibility that can be expected in regards to keeping in contact outside working hours. The overall theme in the studies reviewed was however that flexibility generally seemed to be perceived as valuable.

The main focus of this review was to assess strategies used by peer supporters to deliver interventions based on home visit. However, what is delivered does not always correspond with what is planned. When implementing interventions of this kind it is important to be attentive to this potential disparity, and to evaluate not only the outcomes of the intervention but also the process of implementation. Process evaluation involves, among other things, assessing the quantity and quality of what is delivered, and fidelity to the intervention plan. While most of the studies included descriptions of intended frequency of contact, few of them assessed what dose the individual participants ended up receiving. This is especially important to evaluate for interventions with a high level of flexibility regarding intensity and durations of visits, as the dose received may affect the effectiveness of the intervention. Equally important to reflect upon is the quality of what is delivered, as different families may receive home visits that are delivered differently. The potential discrepancy between plan and practice was generally not thoroughly described or discussed in the studies.

In general, the included studies included only brief descriptions of what the training of the peer supporters entailed, and training was not part of the synthesis of this review. As many of the programs were aimed towards families with multiple social risk factors, the training of the peer supporters has to reflect this complexity. Future reviews of the literature may want to explore how the set-up of training in these programs relates to outcomes for families with health complexity.

All in all, peer support seems able to complement rather than replace social and health care through filling a gap in the provision of empathic support and offering solutions to problems in everyday life. Such support may be provided through the use of several conceptually different strategies, and both supporters and supported parents reported positive experiences of a multitude of intervention programs.

### Limitations

The search strategy used to find studies for this review was adapted to find articles using many different denominations for peer supporters. The lack of standardized terminology may however have affected what studies could be found through this search. Another limitation was that all included studies were set in the U.S. and the U.K., and this relative homogeneity of settings may have had an impact on the types of strategies used to provide support.

## Conclusion

Disadvantaged families often have higher needs in regards to parental and child health, and home-visiting interventions delivered by peer supporters have been used and evaluated in several high-income settings. The strategies used to initiate contact and engage participants could be classified into a number of distinct categories, displaying similarities and variations in how interventions were designed.

## Supplementary information

**Additional file 1.**

## Data Availability

The search strategies used are detailed in the Methods section, and the articles included in this review are listed in Table 1.

## Abbreviations

AOR: Adjusted Odds Ratio
BMI: Body Mass Index
CASP: The Critical Appraisal Skills Programme
CI: Confidence Interval
EPDS: Edinburgh Postnatal Depression Scale
HIV: Human Immunodeficiency Virus
OR: Odds Ratio
RR: Risk Ratio
